# Forcing vine regrowth under different irrigation strategies: effect on polyphenolic composition and chromatic characteristics of cv. Tempranillo wines grown in a semiarid climate

**DOI:** 10.3389/fpls.2023.1128174

**Published:** 2023-05-09

**Authors:** Nieves Lavado Rodas, David Uriarte Hernández, Daniel Moreno Cardona, Luis A. Mancha Ramírez, María Henar Prieto Losada, María Esperanza Valdés Sánchez

**Affiliations:** ^1^CICYTEX-INTAEX, Technological Institute of Food and Agriculture of Extremadura, Badajoz, Spain; ^2^CICYTEX-FOV, Agricultural Research Institute Finca La Orden-Valdesequera, Crta. A-V, Badajoz, Spain

**Keywords:** anthocyanin, catechin, tannin, malvidin, petunidin, delphinidin, peonidin, cyanindin

## Abstract

One of the effects of climate change in warm areas is the asynchrony between the dates of the technological and the phenolic maturity of grapes. This is important because the quality and color stability of red wines are directly related to the content and distribution of phenolic compounds. A novel alternative that has been proposed to delay grape ripening and make it coincide with a seasonal period more favorable for the formation of phenolic compounds is crop forcing. This consists of severe green pruning after flowering, when the buds of the following year have already differentiated. In this way, the buds formed during the same season are forced to sprout, initiating a new delayed cycle. The aim of the present work is to study the effect on the phenolic composition and color of wines elaborated from vines fully irrigated (C), grown using conventional non-forcing (NF) and forcing (F) techniques (C-NF and C-F), and wines from vines subjected to regulated irrigation (RI), grown using NF and F techniques (RI-NF and RI-F). The trial was carried out in an experimental vineyard of the Tempranillo variety located in a semi-arid area (Badajoz, Spain) in the 2017–2019 seasons. The wines (four by treatment) were elaborated and stabilized according to the classic methodologies for red wine. All wines had the same alcohol content, and malolactic fermentation was not carried out in any of them. Anthocyanin profiles were analyzed by HPLC, and total polyphenolic content, anthocyanin content, catechin content, the contribution to color due to co-pigmented anthocyanins, and various chromatic parameters were also determined. Although a significant effect of year was found for almost all the parameters analyzed, a general increasing trend in F wines was found for most of them. The anthocyanin profile of F wines was found to differ from that of C wines, especially in delphinidin, cyanidin, petunidin, and peonidin content. These results indicate that by using the forcing technique it was possible to increase the polyphenolic content by ensuring that the synthesis and accumulation of these substances occurred at more suitable temperatures.

## 1 Introduction

The climate is a determining factor of the physico-chemical characteristics of grapes and, consequently, of the wines obtained (Mira de Orduña, 2010). Air temperature, thermal oscillation, CO_2_ content in the air and solar radiation all play an essential role, as they condition the processes of synthesis, transport, and accumulation of primary and secondary metabolites during grape ripening (Van Leeuwen and Destrac-Irvine, 2017; Van Leeuwen et al., 2019; Jones et al., 2022). In recent decades and in many regions, the previous balance between the above factors has been upset by the changes that have been occurring to the climate as a result of global warming and the associated temperature increases over ever lengthening periods, more sever and longer periods of drought, and increases in CO_2_ and ultraviolet radiation (Sadras and Moran, 2012; Moriondo et al., 2013).

It is known that high temperatures accelerate the accumulation of total soluble solids (TSS) (Petrie and Sadras, 2008), affect the content of acids present in the berries, and modify the content and distribution in grapes of phenolic compounds (Tate, 2001; Monagas et al., 2005; Downey et al., 2006; Rienth et al., 2016; Arrizabalaga-Arriazu et al., 2020). It has also been reported that secondary metabolites in *Vitis vinifera* L. cv. Tempranillo grapes are influenced by ultraviolet radiation, affecting berry development (Del-Castillo-Alonso et al., 2021)

The degree of grape maturity at harvest time is one of the most important parameters for obtaining high-quality red wines. Under current climatic conditions, the grapes reach high TSS values and very low total acidity (TA) before achieving phenolic maturity. That is, technological maturity (adequate concentrations of sugars and acids) and phenolic maturity (determined by the optimal concentration and profile of phenolic compounds) are not simultaneously reached. These changes affect the microbiology process in winemaking progress, as well as the physico-chemical composition and organoleptic characteristics of the wine (Jones et al., 2005; Mira de Orduña, 2010; Palliotti et al., 2014), leading to the production of unbalanced wines with high alcohol content, low acidity, a modified varietal aroma, and a lack of color (Martinez De Toda et al., 2019).

Soil water availability is also a critical factor for vine performance and wine composition (Intrigliolo and Castel, 2010; Afifi et al., 2021; Lizama et al., 2021; Valdés et al., 2022). In arid and semi-arid environments, irrigation is a major tool used to regulate the availability of soil water to vines. Due to the increase in periods of drought and the decreasing availability of water, regulated deficit irrigation (RDI) has become a widely used strategy to reduce the possible negative impact of irrigation on grapes, improving grape composition, and resulting in water savings (Pérez-Álvarez et al., 2021). Deficit irrigation consists in applying water rates to replace only part of the potential vine evapotranspiration either throughout the season or only during specific and previously established phenological periods to control growth and reproductive development and/or improve water use efficiency (Intrigliolo and Castel, 2010). In previous studies, when compared with conventional irrigation RDI techniques resulted in modification of the anthocyanin profile, increasing the anthocyanin content of the grapes and the corresponding wines and improving the sensory characteristics of cv. Tempranillo from vines grown in different semi-arid climate regions of the Iberian Peninsula (Valdés et al., 2009; Intrigliolo and Castel, 2010; Intrigliolo and Castel, 2011; Santesteban et al., 2011; Gamero et al., 2014; Torres et al., 2021). In addition, it has been found that the effect of RDI is highly dependent on the timing of the water deficit (Girona et al., 2009; Valdés et al., 2009; Intrigliolo et al., 2012; Valdés et al., 2022).

In order to maintain and/or improve the characteristics of the wines typical of each area, as well as to preserve the viability of vineyards, it is necessary to adapt them to the new climatic conditions. Such adaptation commonly involves decisions concerning vineyard topographic variables, as well as rootstock, variety, clone, training system, row orientation, and slope selection (Torres et al., 2017; Díaz-Fernández et al., 2022; Gutiérrez-Gamboa et al., 2020; Muñoz-Organero et al., 2022).

Today, short-term viticulture strategies are also being investigated which comprise the establishment of techniques capable of delaying grape ripening and, therefore, shifting harvest date to periods of more favorable temperatures. One such recently proposed technique is crop forcing which consists of severe green pruning after flowering when the buds of the following year have already differentiated. In this way, the flowering of the buds formed that same season is forced, and the whole phenological cycle is delayed, including the beginning of the ripening cycle and the harvest date. This technique was studied by Gu et al. (2012) who managed to significantly delay the harvest date significantly, while other authors have achieved similar results (Lavado et al., 2019; Martinez De Toda et al., 2019; Martínez-Moreno et al., 2019; Kishimoto et al., 2022). While these studies showed lower yield and increased acidity and phenolic compound values as a result of the use of this technique, little is still known about the effects of crop forcing and its interaction with irrigation strategies on the acid, phenolic, and chromatic characteristics of the resulting wines. Other questions that require further research include how the effect of crop forcing changes depending on the meteorological conditions of the year in question, as well as the long-term effect of the technique.

The aim of the work presented here was to study for three consecutive years the effect of crop forcing and its interaction with a pre-veraison RDI strategy on the physico-chemical characteristics, with particular emphasis on the anthocyanin profile, of the resulting wines. An additional aim was to observe the influence of different meteorological circumstances on the effect of the techniques applied.

## 2 Materials and methods

### 2.1 Location and description of the vineyard

The study was carried out in an experimental vineyard located at Badajoz, Extremadura, Spain (38°C 51´ N; 6° 40´ W; 198 m) in a cv. Tempranillo vineyard (*Vitis vinifera* L.) with vines grafted on Richter 110 rootstock and trained as bilateral cordons in a vertical trellis system with a drip irrigation system of 8 L/h per vine. All vines were winter pruned to six spurs and two buds per spur. The rows are E-W oriented and row and vine spacing were 2.5 m and 1.2 m, respectively. The soil is alluvial, with a loam to sandy texture, slightly acidic, and lacking in organic matter. Soil depth is greater than 2.5 m and with low stone content.

### 2.2 Treatments and experimental design

The experimental design was a split-plot with four replications (**Table 1**). The main factor was pruning with two treatments, one with crop forcing technique (F) applied 22 days after anthesis (May 18, 2017; May 29, 2018; May 20, 2019), the other without crop forcing techniques (NF) with vines grown under conventional practices (just winter pruning). Crop forcing consisted of hedging the growing shoots to seven nodes and removing all the summer laterals, leaves, and clusters with scissors to force the bursting of the primary buds developed in the current season. As a secondary factor, two irrigation treatments were established: a treatment with no water stress (C), supplying water to maintain a pre-established midday stem water potential (SWP), and a pre-veraison deficit irrigation (RI).

**Table 1:** Summary of the treatments applied in the study.

Treatment	No crop forcing (NF)	Crop forcing (F)
Fully irrigated (C)	C-NF	C-F
Deficit irrigation (RI)	RI-NF	RI-F

Vine water requirements were calculated based on the crop evapotranspiration (ETc) using the crop coefficient (Kc) recommended by FAO for these latitudes for the NF treatments. For the F treatments, ETc was calculated directly with a weighing lysimeter (Picón-Toro et al., 2012) for two crop forcing vines, integrated in the study plot. Irrigation started when a threshold SWP value of -0.6 MPa was reached. Irrigation was applied five to six times per week, measuring the amount of water applied to each subplot through volumetric water meters, and maintaining irrigation until the beginning or middle of October. The experimental unit consisted of six rows per 18 vines. The 10 central vines of the four central rows were used for sampling.

### 2.3 Weather conditions

**Figure 1:**
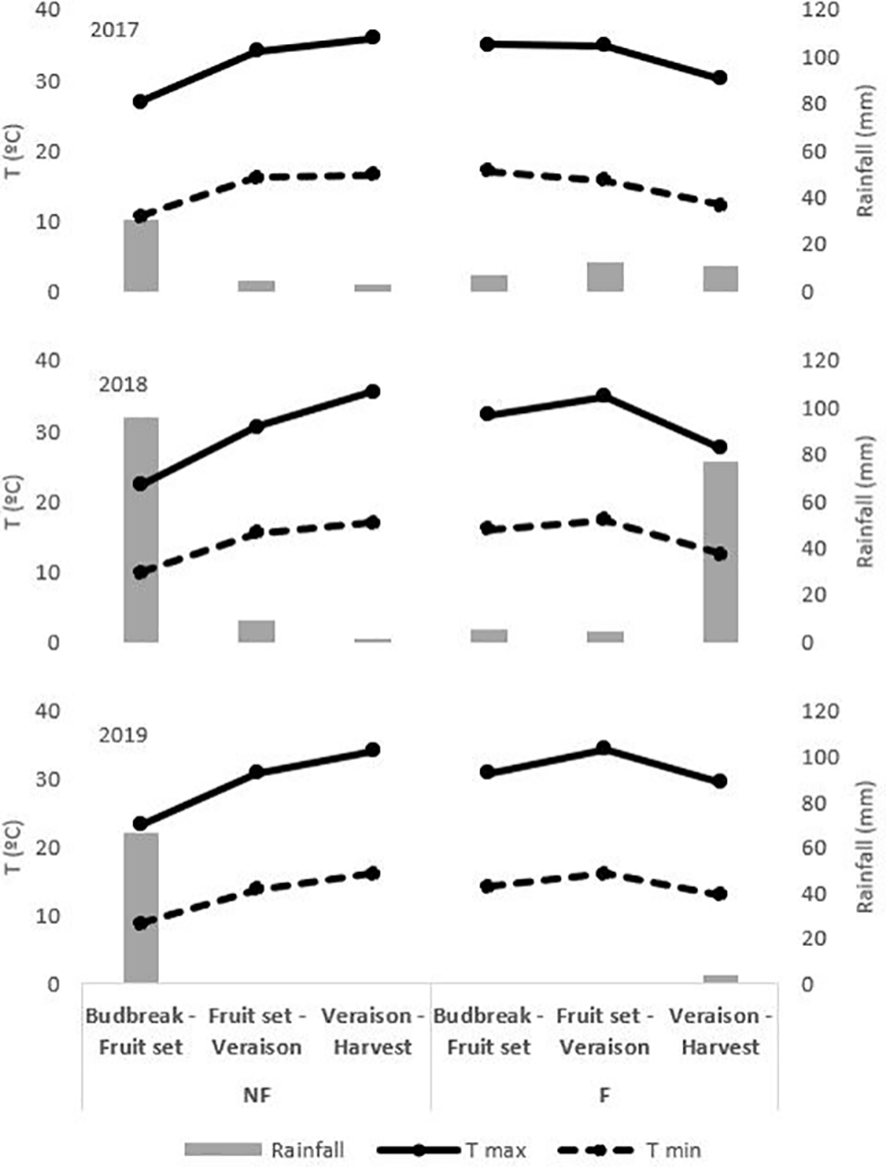
Meteorological conditions on budburst to fruit set, fruit set to veraison and veraison to harvest period on NF and F treatments in 2017, 2018 and 2019. *Treatments: NF (no forcing) and F (forcing)*.

The area has a Mediterranean climate with a mild Atlantic influence, dry and hot summers, with high daily radiation and evaporative demand. The meteorological data was obtained from a weather station belonging to the Extremadura Irrigation Advisory Network (REDAREX for its initials in Spanish) located 100 m from the plot, with the characteristics described in Martí et al. (2015). **Figure 1** shows the meteorological conditions (T max, med and min and rainfall) in the budburst to fruit set, fruit set to veraison, and veraison to harvest periods for the different treatments and the 3 years of trial.

### 2.4 Vine phenology

Phenology monitoring was performed weekly according to the modified E-L system (Coombe, 1995). Starting from mid-March (“cotton bud” stage), a visual inspection was made of 10 plants per plot to determine the most representative growth stage (the stage shown by at least 50% of plants) as well as the most backward and the most advanced stages in the samples. **Table 2** shows the day of year of the different phenological states from budbreak to harvest in the different treatments (Lavado et al., 2023).

**Table 2:** Day of the year for the different phenological stages in each treatment and day of application of crop forcing during the 2017, 2018 and 2019 seasons (Lavado et al., 2023).

Phenological stage	2017		2018		2019	
	NF	F	NF	F	NF	F
Budbreak	93	93	93	99	85	91
Crop forcing		157		168		154
Fruit set	150	201	156	214	140	200
Veraison	187	256	204	261	200	253
Harvest	234	292	241	302	240	283

Treatments: NF (no forcing) and F (forcing). Each treatment represents the two irrigation regimes as no differences were found between them.

### 2.5 Water status

The SWP was measured with a pressure chamber (Soil Moisture Corp., Model 3500, Santa Barbara, CA, USA), following the procedure described by Shackel (2007), using leaves on the north side of the trellis (in the shade), close to trunk level and wrapped in aluminum foil at least 2 h before data recording. Measurements were taken weekly on one leaf per vine and in two plants per subplot. **Table 3** shows the average midday SWP (MPa) throughout the different phenological stages in 2017, 2018, and 2019 (Lavado et al., 2023).

**Table 3:** Average midday stem water potential (MPa) throughout the different phenological stages in 2017, 2018 and 2019.

Year	Phenological Stage	Treatment			
		C-NF	C-F	RI-NF	RI-F
**2017**	**BB-CFP**		−0.63		−0.62
	**CFP-F**	−0.49	−0.55	−0.56	−0.69
	**F-FS**	−0.64	−0.79	−0.72	−1.03
	**FS-V**	−0.61	−0.74	−1.12	−0.9
	**V-H**	−0.82	−0.58	−1.24	−0.71
**2018**	**BB-CFP**		−0.49		−0.51
	**CFP-F**	−0.41		−0.48	
	**F-FS**	−0.41	−0.62	−0.48	−0.78
	**FS-V**	−0.49	−0.61	−1.00	−0.96
	**V-H**	−0.61	−0.59	−0.92	−0.67
**2019**	**BB-CFP**		−0.64		−0.73
	**CFP-F**				
	**F-FS**	−0.65	−0.58	−0.77	−0.58
	**FS-V**	−0.70	−0.64	−1.06	−0.78
	**V-H**	−0.75	−0.62	−0.78	−0.77

Treatments: C-NF (no forcing and full irrigation); C-F (forcing and full irrigation); RI-NF (no forcing and deficit irrigation) and RI-F (forcing and deficit irrigation). BB, Budbreak; CFP, Crop forcing pruning; F, Flowering; FS, Fruit set; V, Veraison; H, Harvest (Lavado et al., 2023).

### 2.6 Yield parameters

All treatments were harvested manually at 23–24°Brix, a common criterion for picking red grape varieties in the study area. The average TSS of the berries from the four elementary plots was considered for each treatment. To determine the effects of the treatments on yield parameters and berry weight, all the clusters of 10 vines per experimental plot were weighed (40 vines per treatment) and samples of 100 g of berries per plot were weighed fresh.

### 2.7 Microvinifications

Each year, 16 microvinifications were carried out (4 treatments × 4 experimental blocks) according to the following experimental protocol: samples of about 60 kg were destemmed and mechanically crushed (Modelo Micra/15, Agrovin, Spain). One aliquot (100 g) of initial mash (pulp, juice, skins and seeds) was frozen (-80°C) for the analysis of polyphenol compounds. On the other hand, 50 mL of must were taken, filtered and immediately analyzed. The mash was fermented in 50-L steel tanks at 22–24°C. Initially, sulphur dioxide (SO_2_) was added at 50 mg/kg and *Saccharomyces cerevisiae* (Viniferm 3D, Agrovin, Spain) were inoculated (25 g/hL). Fermentation was monitored daily, measuring density and total phenolic index (TPI) by spectrophotometric absorbance at 280 nm (UV/visible UV-1700 spectrophotometer, Shimadzu, Shimadzu Corporation, Kyoto, Japan). During vatting, fermenting must wines were punched twice per day. The musts were racked when the increase in TPI leveled off. Once fermentation was completed, the wines were settled at 4°C, and sulphur was then added to the wine to achieve 30 mg/L of free SO_2_. Finally, the wines were bottled and stored at 15°C until analysis, without initiating malolactic fermentation. Thus, 16 different wines were obtained (4 treatments × 4 blocks per treatment).

### 2.8 Analytical methods

#### 2.8.1 General oenological parameters

The TSS (°Brix) of musts was determined by refractometry (RE40D, Mettler Toledo, Greifensee, Switzerland). Wine analysis was carried out 4 months after bottling. In musts and wines, analysis for pH and TA (g tartaric acid/L) was conducted following the OIV (Organisation Internationale de la Vigne et du Vin) methods (1990) in an automatic titrator (T50, Mettler Toledo, Greifensee, Switzerland) according to ECC formal methods (ECC, 1990). Tartaric acid (TAR, g/L) and malic acid (MAL, g/L) were analyzed following the Rebelein and the enzymatic reaction methods, respectively, in an autoanalyzer (Y15, Biosystems, Barcelona, Spain).

#### 2.8.2 Nitrogen parameters

Free amino nitrogen (FAN, mg/L) and ammonium (NH_4_^+^, mg/L) content were enzymatically analyzed according to ECC formal methods (ECC, 1990) using an autoanalyzer (Y15, Biosystems, Barcelona, Spain).

#### 2.8.3 Phenolic compounds

The extraction of phenolic substances from the initial mash was carried out following a methodology based on previous works (Portu et al., 2016; Díaz-Fernández et al., 2022). An aliquot (1.0 g) of the frozen mash was homogenized for 30 s in a Freshboost blender (LM180110, Moulinex, Aleçon, France) with 10 mL of a hydroalcoholic solution (methanol/water/formic acid 50:48.5:1.5, v/v/v) and then the mixture was macerated (30 min, 4°C, ultrasonic bath USC-TH, VWR, Radnor, USA) and centrifuged (4°C, 10 min 5810 R, Eppendorf, Hamburg, Germany). The supernatant was separated, and the resulting pellet was reextracted up to three times. The supernatants (phenolic extracts) were combined and the final volume was annotated. In the polyphenolic extracts obtained, total polyphenol content (TPP) was determined according to Singleton and Rossi (1965), and total anthocyanin (Ant) content by the pH differential method (Lee et al., 2005) using an autoanalyzer (Y15, Biosystems, Barcelona, Spain). Ant was considered as the sum of substances quantified by HPLC. Tannin content (Tan) was determined using the spectrophotometric method following Sarneckis et al. (2006). Catechin content (Cat) was determined according to Broadhurst and Jones (1978), and co-pigmented anthocyanin (%C-Ant) using the colorimetric effects of acetaldehyde and SO_2_ on different forms of anthocyanins (Boulton, 2001).

#### 2.8.4 Wine chromatic characteristics

Color intensity (CI) was calculated as the sum of absorbance at 420, 520, 620 nm, and hue as the ratio of the absorbance at 420 nm and 520 nm. From absorbance values at 420, 520, and 620 nm, the percentages of yellow (Yellow %), red (Red %) and blue (Blue %) were determined according to the Glories method (Glories, 1984): Yellow % = 100% [Abs 420/(Abs 420 + Abs 520 + Abs 620)]; Red % = 100% [Abs 520/(Abs 420 + Abs 520 + Abs 620)]; Blue % = 100% [Abs 620/(Abs 420 + Abs 520 + Abs 620)]. Absorbance measurements were taken using a Shimadzu spectrophotometer with data system control software (Shimadzu Corporation, Kyoto, Japan). Samples were filtered through Millipore-AP20 filters (Bedford, MA, USA) prior to color determination.

#### 2.8.5 Anthocyanin compounds by HPLC

HPLC separation, identification and quantification of anthocyanins were performed on an Agilent 1200 LC system (Agilent Technologies, Palo Alto, CA) equipped with a degasser, quaternary pump, column oven, 1290 infinity autosampler, UV-Vis diode-array detector (DAD) and the Chemstation software package for LC 3D systems (Agilent Technologies) to control the instrument and for data acquisition and analysis. Separation was performed in a Kromasil^®^ 100-5-C18 (250 × 4.6 mm) column (AkzoNobel, Bohus, Sweden). The analysis was carried out as described in Natividade et al. (2013) with slight modifications to improve peak resolution. For the analysis of anthocyanins, a 10 mL extract was injected directly into the HPLC and the column was maintained at 40°C. The mobile phase consisted of a gradient mixture of a solvent A (0.85% phosphoric acid solution) and solvent B (acetonitrile), with a flow rate of 1 mL·min^-1^. The gradient was started with 100% of solvent A and adjusted for 90% of solvent A and 10% of solvent B at 10 min; 85% of solvent A and 15% of solvent B at 20 min; 80% of solvent A and 20% of solvent B at 30 min; 67% of solvent A and 33% of solvent B at 40 min; 65% of solvent A and 35% of solvent B at 45 min; and 100% of solvent B at 55 min. Absorbance at 520 nm was measured by the DAD detector for identification of anthocyanins by their elution order and by comparison to the retention times of commercially available standards (malvidin-3-glucoside, delphinidin-3-O-glucoside, cyanidin-3-O-glucoside, and peonidin-3-O-glucoside (Extrasynthese, Genay, France). The anthocyanins present in the wines were identified as the mono-glucoside forms (G) of malvidin (MvG), petunidin (PtG), delphinidin (DpG), peonidin (PnG) and cyanidin (CyG); the acetyl-glucoside forms (MvA, PtA, DpA, PnA, and CyA), and the p-coumaroyl-glucoside forms (MvC, PtC, PnC, and CyC). All measures were expressed in mg glucoside/L.

### 2.9 Statistical data analysis

Normality and homogeneity of variances were tested using the Shapiro-Wilk’s and Bartlett’s test respectively. When the normality and homogeneity of variances were verified, yield and berry weight data were analyzed by one-factor analysis of variance (ANOVA) and the Tukey test. Differences between means were considered statistically significant when *p* < 0.05. Must and wine data were subjected to multiple analyses of variance (MANOVA) to investigate the effect of “crop forcing”, “irrigation” and their interaction on each parameter evaluated, selecting *p* ≤ 0.001, *p* ≤ 0.01, and *p* ≤ 0.05 for significance of comparisons. The interaction between effects was evaluated by calculating the least-squares means (LS means) selecting *p* ≤ 0.001, *p* ≤ 0.01, and *p* ≤ 0.05 for significance of comparisons and the Tukey test as *post hoc* test for parametric samples. When normality and homogeneity of variances were not verified, non-parametric tests were carried out and the Kruskal-Wallis test (alternative to one-way ANOVA) and multiple comparison p values (alternative to *post-hoc* pairwise comparisons) were used. Differences between means were considered statistically significant when *p* < 0.05. Multiple factor analysis (MFA) was applied. Meteorological conditions were used as supplementary variable, with yield and acid and phenolic composition and chromatic characteristics of the resulting wines as active variables. Principal component analysis (PCA) was performed to discriminate between treatments on the basis of the values of Mv, Pt, Dp, Pn, and Cy. These last statistical tests were performed with XLSTAT-Pro statistical software package (Addinsoft, 2009, Paris, France).

## 3 Results

The effect of the year on agronomical parameters and all the must and wine composition parameters studied was highly significant (data not shown), suggesting that the effect of treatments, especially that caused by the irrigation regime, on these parameters was different between seasons. For this reason, the results of each harvest are analyzed separately in this work. To minimize compositional differences between treatments associated with TSS level, a common TSS between treatments and year was considered.

### 3.1 Yield parameters

**Table 4** shows the yield (kg/vine) and berry weight (BW, g) of the control treatment (C-NF) in the 2017–2019 period. Yields were lower than 4 kg/vine in 2017 and 2018 and close to 7 kg/vine in 2019 in the control treatment (C-NF). Significantly lower values were found each year in the F compared to the NF treatments. In 2017, the yield penalty was higher in RI-F (66%) than in C-F (29%), while in 2018 and 2019 the values were similar and close to 50% and 60%, respectively. In RI-NF, the loss of yield was 42%, 18% and 38% in 2017, 2018 and 2019 with respect to C-NF. The decrease in 2017 in this treatment was higher than in C-F, in contrast to what was observed in the following years. Regarding BW, in both forcing and irrigation treatments significant decreases were observed with respect to C-NF. The following BW sequence was observed: RI-F > C-F > RI-NF.

**Table 4:** Effect of crop forcing and water status treatment on yield (kg/vine) and berry weight (g).

Parameter	Year	C-NF values	% Decrease in harvest compared to C-NF				*FxI*	NF values	% Decrease in harvest compared to NF		*F*	C values	% Decrease in harvest compared to C		*I*
			C-NF	C-F	RI-NF	RI-F			NF	F			C	RI	
Yield Kg/ha	2017	3.9	0	−29	−42	−66	*n.s.*	3.1	0	−34	***	3.3	0	−46	***
	2018	3.7	0	−51	−18	−52	*n.s.*	3.4	0	−47	***	2.8	0	−13	*
	2019	7.1	0a	−58 c	−38 b	−60 c	***	5.8	0	−49	***	5.1	0	−28	***
Berry weight (g)	2017	1.8	0a	−44 c	−28 b	−56 c	*	1.6	0	−42	***	1.4	0	−25	***
	2018	1.9	0a	−37 b	−32 b	−47 b	*	1.6	0	−31	***	1.6	0	−26	***
	2019	2.3	0a	−39 b	−35 b	−48 b	**	1.9	0	−32	***	1.9	0	−27	***

Treatments: C-NF (no forcing and full irrigation); C-F (forcing and full irrigation); RI-NF (no forcing and deficit irrigation) and RI-F (forcing and deficit irrigation); F, Forcing factor; I, Irrigation factor.

Each value represents the mean of 8 samples (4 blocks, 2 replicates). Statistical analysis: Different letters indicate the existence of statistically significant differences between treatments; n.s. indicates not significant; (*) significant at 5% level; (**) significant at 1% level and (***) significant at 0.1% level.

### 3.2 Must composition

**Table 5** shows the acid, nitrogen and phenolic composition of the initial musts and mash from the different treatments applied to the vines and the results of the MANOVAs performed to analyze the statistical significance of the effect of crop forcing (F), irrigation treatment (I), and their interaction on these parameters in 2017, 2018 and 2019. In general, no statistical significance was observed in the *FxI* interaction (only on 3 occasions). Although the statistical significance of both F and I depended on the parameter analyzed and the year considered, it should be noted that crop forcing had a more significant number of times of statistical significance (18) than irrigation treatment (10). Musts showed similar TSS values for each year and treatment. Regarding acid composition, the results shown in **Table 5** do not allow general conclusions to be drawn on the effect of F on TAR because high variability between years was observed. In 2017, an *FxI* interaction was detected because F caused a significant increase (12.0%, *p* < 0.05) in the contents of the C musts (C-F vs. C-NF), while no effect was detected in RI. In 2018, all musts presented similar values. Finally, when F must was compared with NF must in 2019, similar decreases were found regardless of irrigation treatment. With respect to the crop forcing treatment, irrigation treatment did not have any effect in any year. As **Table 5** shows, crop forcing increased MAL values, with higher increases in RI treatments. The increases in 2017, 2018, and 2019 were 267%, 53%, and 105% in RI-F vs. RI-NF, but 30% and 6% and 71% in CF vs. C-NF. In 2017, a significant *FxI* interaction was found because the C-NF value was much higher than the RI-NF (233% increase), while C-F had a similar value to RI-F. In 2018, C-NF and C-F were higher than RI-NF and RI-F, respectively. Finally, in 2019 similar values were found in C and RI must. As a consequence of these results, the RI-NF musts had the lowest MAL values in the three years of the trial. The variations in TAR and especially MAL values modified the TA values. Thus, in 2017 and 2019, higher TA values were observed in the F than the NF musts (*p* < 0.001 in 2017 and *p* < 0.01 in 2019). In 2018, and as a logical consequence of the values of both MAL and TAR, the C-NF and C-F musts presented similar TA values (7.0 and 7.3, respectively), but the RI-NF value (4.8) differed considerably from that of RI-F (6.0). Moreover, in all years TA of the RI musts was significantly lower than for the corresponding C musts. Because of these results, the C-F and RI-NF musts respectively had the highest and lowest TA values in the three years of the study. Finally, in the 2017 and 2018 vintages, the pH of the F musts was significantly lower than that of the NF musts, and the RI musts, especially the NF ones, showed higher pH values than the corresponding C musts. Consequently, in those vintages, the highest pH values corresponded to RI-NF musts (3.9 and 3.7 in 2017 and 2018) and the lowest to C-F musts (3.6 and 3.4 in those same years). However, in 2019, all musts presented similar pH values.

**Table 5:** Effect of crop forcing and water status on composition of cv. Tempranillo musts.

Parameter	Year	Treatment										
		C-NF	C-F	RI-NF	RI-F	*FxI*	NF	F	*F*	C	RI	*I*
**TSS** **(°Brix)**	**2017**	24.8	23.7	24.0	23.2	*n.s.*	24.4	23.5	*n.s.*	24.3	23.6	*n.s.*
	**2018**	25.2	24.1	23.8	22.8	*n.s.*	24.5	23.5	*n.s.*	24.7	23.3	*n.s.*
	**2019**	22.5	23.2	23.6	23.9	*n.s.*	23.0	23.5	*n.s.*	22.8	23.7	*n.s.*
*Acid composition*												
**TAR** **(g/L)**	**2017**	3.9 b	4.4 a	4.1 ab	4.2 ab	*	4.0	4.3	**	4.2	4.2	*n.s.*
	**2018**	4.6	4.8	4.5	4.4	*n.s.*	4.5	4.6	*n.s.*	4.7	4.5	*n.s.*
	**2019**	5.0	4.6	5.2	4.7	*n.s.*	5.1	4.7	**	4.8	4.9	*n.s.*
**MAL** **(g/L)**	**2017**	2.0 b	2.6 a	0.6 c	2.2 ab	*	1.3	2.4	***	2.3	1.4	***
	**2018**	3.1	3.3	1.9	2.9	*n.s.*	2.5	3.1	*	3.2	2.4	**
	**2019**	2.1	3.6	1.8	3.7	*n.s.*	2.0	3.6	***	2.8	2.8	*n.s.*
**TA** **(g/L)**	**2017**	4.4	5.6	3.7	4.9	*n.s.*	4.1	5.3	***	5.0	4.3	**
	**2018**	7.0	7.3	4.8	6.0	*n.s.*	5.9	6.7	*n.s.*	7.1	5.4	**
	**2019**	6.8	7.2	5.3	7.0	*n.s.*	6.1	7.1	**	7.0	6.2	*
**pH**	**2017**	3.9	3.6	3.9	3.6	*n.s.*	3.9	3.6	***	3.7	3.7	*n.s.*
	**2018**	3.5	3.4	3.7	3.4	*n.s.*	3.6	3.4	**	3.4	3.5	*
	**2019**	3.4	3.5	3.6	3.5	*n.s.*	3.5	3.5	*n.s.*	3.4	3.5	*n.s.*
*Nitrogen composition*												
**NH_4_^+^** **(mg/L)**	**2017**	125.0	71.8	121.8	68.0	*n.s.*	123.4	69.9	***	98.4	94.9	*n.s.*
	**2018**	81.8	40.9	93.8	47.7	*n.s.*	87.8	44.3	**	61.3	70.7	*n.s.*
	**2019**	67.7	58.2	82.0	54.5	*n.s.*	74.8	56.3	*n.s.*	62.9	68.3	*n.s.*
**FAN** **(mg/L)**	**2017**	148.3	68.8	141.0	62.8	*n.s.*	144.6	65.8	***	108.5	101.9	*n.s.*
	**2018**	70.3 b	52.8 b	100.5 a	53.3 b	*	85.4	53.1	***	61.5	76.9	*
	**2019**	57.7	57.0	91.0	56.4	*n.s.*	74.3	56.7	*n.s.*	57.3	73.7	*n.s.*
*Phenolic composition*												
**Ant** **(mg/L)**	**2017**	918.3	982.8	1212.1	1286.3	*n.s.*	1065.2	1134.5	*n.s.*	950.6	1249.2	**
	**2018**	916.1	1222.7	956.6	1436.9	*n.s.*	936.4	1329.8	***	1069.4	1196.8	*n.s.*
	**2019**	705.5	1138.8	867.0	1264.8	*n.s.*	786.2	1201.8	**	922.1	1065.9	*n.s.*
**Tan** **(mg/L)**	**2017**	4738.4	5041.9	4385.4	4743.4	*n.s.*	4561.9	4892.7	*n.s.*	4890.2	4564.4	*n.s.*
	**2018**	4932.5	6120.7	4105.2	4211.2	*n.s.*	4518.8	5166.0	*n.s.*	5526.6	4158.2	**
	**2019**	4436.1	5671.3	4588.6	5199.6	*n.s.*	4512.4	5435.4	**	5053.7	4894.1	*n.s.*
**TPP** **(mg/L)**	**2017**	4934.3	5155.2	4552.1	5106.9	*n.s.*	4743.2	5131.1	*n.s.*	5044.8	4829.5	*n.s.*
	**2018**	4928.5	6741.2	4235.3	5361.6	*n.s.*	4581.9	6051.4	**	5834.8	4798.4	**
	**2019**	3392.6	5328.1	3759.7	5030.4	*n.s.*	3576.1	5179.2	***	4360.4	4395.0	*n.s.*

Treatments: C-NF (no forcing and full irrigation); C-F (forcing and full irrigation); RI-NF (no forcing and decit irrigation) and RI-F (forcing and deficit irrigation); F, Forcing factor; I, Irrigation factor.

Parameters: Total soluble solids (TSS), Tartaric acid (TAR), Malic acid (MAL), Total acid (TA), pH, Free amino nitrogen (FAN), Ammonium (NH_4_^+^), Anthocyanins (Ant), Tannins (Tan) and Total polyphenolics (TPP).

Each value represents the mean of 8 samples (4 blocks, 2 replicates). Statistical analysis: Different letters indicate the existence of statistically significant differences between treatments; n.s. indicates not significant; (*) significant at 5% level; (**) significant at 1% level and (***) significant at 0.1% level.

Regardless of the treatment considered, it is noteworthy that as the study progressed, a gradual decrease in the values of nitrogen compounds (both inorganic and organic) was observed. In 2017 and 2018, the NH_4_^+^ values of the F musts were lower than those of the NF musts. In both irrigation strategies, the decreases were around 50%. In 2019 no differences were found. In none of the years did the use of RDI cause significant changes in the values of this parameter. Finally, the free amino nitrogen (FAN) contents of the musts were modified in a very similar way to those of NH_4_^+^, and thus the mean values of this parameter of the F musts in 2017 and 2018 (144.6 and 85.4) were significantly lower than those recorded in the NF musts (65.8 and 53.1).

The Ant, Tan, and TPP results shown in **Table 5** indicate that crop forcing caused, in both C and RI, a general trend to higher values, with significant increases of Ant and TPP in 2018 and 2019 and Tan in 2019 only. The changes due to irrigation strategy were less significant. In fact, compared to C, significant increases were only observed in RI in 2017 in the Ant values, and in 2018 decreases in Tan and TPP.

### 3.3 Wine composition

**Table 6** shows the effects of treatments on the alcohol content (AD, % v/v), acid composition, phenolic composition and chromatic characteristics of the wines from 2017, 2018, and 2019. In 2017 and 2018, all wines showed similar AD values, while in 2019, contrary to expected, since the initial musts had similar TSS values, the AD of the C-F (12.8) and RI-F (13.2) wines was lower than that of the C-NF (14.0) and RI-NF (14.2) wines.

**Table 6:** Effect of crop forcing and water status on composition and chromatic characteristics of Tempranillo wines.

Parameter	Year	Treatment										
		C-NF	C-F	RI-NF	RI-F	*FxI*	NF	F	*F*	C	RI	*I*
**AD (%)**	**2017**	14.8	13.8	14.0	13.5	*n.s.*	14.4	13.6	*n.s.*	14.3	13.7	*n.s.*
	**2018**	14.9	14.1	14.3	13.3	*n.s.*	14.6	13.7	*n.s.*	14.5	13.8	*n.s.*
	**2019**	14.0	12.8	14.2	13.2	*n.s.*	14.1	13.0	*	13.4	13.7	*n.s.*
*Acid composition*												
**TAR (g/L)**	**2017**	1.8	2.4	2.2	2.6	*n.s.*	2.0	2.5	**	2.1	2.4	*n.s.*
	**2018**	2.2	2.2	2.3	2.5	*n.s.*	2.3	2.3	*n.s.*	2.2	2.4	*n.s.*
	**2019**	2.4	2.5	2.3	2.5	*n.s.*	2.3	2.5	*n.s.*	2.4	2.4	*n.s.*
**MAL (g/L)**	**2017**	2.0	2.8	1.6	2.7	*n.s.*	1.8	2.8	***	2.4	2.2	*n.s.*
	**2018**	3.2 a	3.1 a	2.5 b	3.1 a	*	2.9	3.1	*n.s.*	3.2	2.8	*
	**2019**	2.1 c	2.9 a	2.4 bc	2.7 ab	*	2.2	2.8	***	2.5	2.6	*n.s.*
**TA (g/L)**	**2017**	5.5	6.8	5.0	6.9	*n.s.*	5.2	6.9	***	6.1	6.0	*n.s.*
	**2018**	7.8 ab	8.2 a	6.2 c	7.5 b	*	7.0	7.8	***	8.0	6.8	***
	**2019**	7.5 a	7.6 a	6.0 b	7.8 a	**	6.8	7.7	**	7.6	6.9	*
**pH**	**2017**	4.1	4.0	3.9	3.9	*n.s.*	4.0	3.9	*n.s.*	4.0	3.9	*n.s.*
	**2018**	3.7	3.7	3.8	3.7	*n.s.*	3.8	3.7	**	3.7	3.8	*
	**2019**	3.5 b	3.6 ab	3.7 a	3.6 ab	*	3.6	3.6	*n.s.*	3.5	3.6	*
Phenolic composition												
**Ant** **(mg/L)**	**2017**	148.2	199.2	227.3	217.5	n.s.	187.8	208.4	n.s.	173.7	222.4	*
	**2018**	280.0	441.6	239.7	373.9	n.s.	259.9	407.7	**	360.8	306.8	n.s.
	**2019**	187.5 c	321.3 a	232.0 bc	288.4 ab	*	209.8	304.9	***	254.4	260.2	n.s.
**Cat (mg/L)**	**2017**	995.7	1752.1	715.8	1655.2	*n.s.*	855.7	1703.6	***	1373.9	1185.5	*n.s.*
	**2018**	1528.3	2837.3	1052.5	1888.9	*n.s.*	1290.4	2363.1	***	2182.8	1470.7	**
	**2019**	1810.8 b	3641.1 a	1149.5 c	3633.6 a	**	1480.2	3637.3	***	2726.0	2391.5	*
**Tan (mg/L)**	**2017**	1330.7	1995.9	951.7	1634.7	*n.s.*	1141.2	1815.3	***	1663.3	1293.2	*
	**2018**	2005.7	1535.6	1526.3	1624.5	*n.s.*	1766.0	1580.0	*n.s.*	1770.7	1575.4	*n.s.*
	**2019**	1871.3 b	2707.0 ab	990.5 c	2983.8 a	*	1430.9	2845.4	***	2289.2	1987.1	*n.s.*
**TPP (mg/L)**	**2017**	1581.9	2212.0	1546.4	2101.3	*n.s.*	1564.1	2156.6	***	1896.9	1823.8	*n.s.*
	**2018**	2228.6	2570.1	1783.4	2233.1	*n.s.*	2006.0	2401.6	***	2399.4	2008.2	***
	**2019**	1855.8	2594.5	1762.0	2581.7	*n.s.*	1808.9	2588.1	***	2225.1	2171.9	*n.s.*
Chromatic characteristics												
**C-Ant (%)**	**2017**	17.8	26.1	22.6	31.9	*n.s.*	20.2	29.0	*	22.0	27.3	*n.s.*
	**2018**	32.3	37.1	33.8	44.1	*n.s.*	33.1	40.6	**	34.7	38.9	*
	**2019**	25.9	40.2	32.3	40.5	*n.s.*	29.1	40.3	***	33.0	36.4	*n.s.*
**CI**	**2017**	3.7	5.8	4.1	6.5	*n.s.*	3.9	6.1	**	4.7	5.3	*n.s.*
	**2018**	13.6	14.8	13.8	15.7	*n.s.*	13.7	15.3	*	14.2	14.8	*n.s.*
	**2019**	8.7	12.9	9.7	14.1	*n.s.*	9.2	13.5	**	10.8	11.9	*n.s.*
**Yellow (%)**	**2017**	33.8	31.8	31.3	30.1	*n.s.*	32.5	31.0	*n.s.*	32.8	30.7	*
	**2018**	31.0	29.4	31.9	28.2	*n.s.*	31.4	28.8	**	30.2	30.1	*n.s.*
	**2019**	28.6 b	29.6 ab	30.3 a	29.4 ab	*	29.5	29.5	*n.s.*	29.1	29.9	*n.s.*
**Red (%)**	**2017**	53.8	57.0	57.4	59.2	*n.s.*	55.6	58.1	*n.s.*	55.4	58.3	*
	**2018**	56.8	61.2	54.4	62.3	*n.s.*	55.6	61.8	***	59.0	58.3	*n.s.*
	**2019**	62.2 a	59.9 ab	58.3 b	60.0 ab	**	60.2	60.0	*n.s.*	61.0	59.2	**
**Blue (%)**	**2017**	12.4	11.2	11.3	10.7	*n.s.*	11.9	10.9	*n.s.*	11.8	11.0	*n.s.*
	**2018**	12.2	9.4	13.7	9.5	*n.s.*	13.0	9.4	***	10.8	11.6	*n.s.*
	**2019**	9.2 b	10.5 ab	11.3 a	10.5 a	**	10.3	10.5	*n.s.*	9.9	10.9	**
**Hue**	**2017**	3.1	2.8	2.7	2.6	*n.s.*	2.9	2.7	*n.s.*	3.0	2.6	*
	**2018**	5.5	4.8	5.9	4.5	*n.s.*	5.7	4.7	**	5.1	5.2	*n.s.*
	**2019**	4.6 b	4.9 ab	5.2 a	4.9 ab	*	4.9	4.9	*n.s.*	4.8	5.1	*

Treatments: C-NF (no forcing and full irrigation); C-F (forcing and full irrigation); RI-NF (no forcing and deficit irrigation) and RI-F (forcing and deficit irrigation); F, Forcing factor; I, Irrigation factor.

Parameters: Alcoholic degree (AD); Tartaric acid (TAR); Malic acid (MAL); Total acid (TA); pH; Anthocyanins (Ant); Catechins (Cat); Tannins (Tan); Total polyphenolics (TPP); Percentage of color due to anthocyanins (C-Ant); Color intensity (CI); % Absorbance 420 nm (Yellow%); % Absorbance 520 nm (Red%); % Absorbance 620 nm (Blue%) and Color tonality (Hue).

Each value represents the mean of 8 samples (4 blocks, 2 replicates). Statistical analysis: Different letters indicate the existence of statistically significant differences between treatments; n.s. indicates not significant; (*) significant at 5% level; (**) significant at 1% level and (***) significant at 0.1% level.

#### 3.3.1 Acid composition

As **Table 6** reflects, the statistical significance of the *FxI* interaction was only observed five times and the effect of *F* and *I* depended on the parameter analyzed and on the year in question. As in the case of musts, crop forcing was statistically significant more times (7) than the irrigation treatment (2).

A general trend to increase was found in the values of TAR and MAL in F compared to NF wines. The significance of the effect on these substances depended on the year considered and, in some cases, on the irrigation strategy. Thus, in 2018 higher values of MAL were registered in RI-F vs. RI-NF, but in 2018 C-F values were similar than C-NF. In general, TA values were higher in F than in NF wines, with the most noticeable effect on the RI wines. In this way, a significant *FxI* interaction was found in 2018 and 2019, because only the value of RI-F wines (7.5 and 7.8 in 2018 and 2019) was higher than the corresponding RI-NF (6.2 and 7.5, respectively). Finally, crop forcing decreased the pH values in 2018 only. Concerning irrigation effect, and in agreement with the trend registered in the musts, no differences were observed in TAR values of C and RI (F and NF) wines in any year, and the differences in MAL values found between C and RI musts were only slightly reflected in the wines, with only the C-NF value being significantly higher than the RI-NF value in 2018. Finally, C wines had higher TA than RI wines in 2018 and 2019, in both NF and F wines in 2018 and 2019 and in 2019 in NF wines only. In consequence, in 2018 the pH of RI wines was higher than C wines (regardless of crop forcing), but in 2019 significant increases were only registered in RI-NF vs. C-NF.

#### 3.3.2 Phenolic composition and chromatic characteristics

As a general trend, no interactions were found in terms of wine phenolic composition, chromatic characteristics or anthocyanin profile between the two techniques applied to the vines (**Tables 6**, **7**). The effect of crop forcing and irrigation can therefore be examined, in most cases, separately. As observed in the initial mash, a general trend to higher values of phenolic substances was found in the F compared to the NF wines (**Table 6**), and significant increases were registered in the values of F wines compared to NF wines in all phenolic substances analyzed each year with the exception of Ant in 2017 and Tan in 2018. It can thus be concluded that crop forcing had a clear and consistent effect over the years and improved the phenolic content of the wines. However, the effect of *I* was less significant (significant differences were found five times only) and less consistent because the statistical significance depended on the year considered and the crop forcing applied. Thus, compared to C-NF and C-F, anthocyanin values in RI-NF and RI-F were higher in 2017 (53% and 9%), lower in 2018 (14.4% and 15.3%), while a significant *FxI* interaction was registered in 2019. In contrast to the effect found in F, a general trend to lower catechins (*p* < 0.01 in 2018, *p* < 0.05 in 2019) and tannins (*p* > 0.05 in 2017) was recorded in the RI compared to the C wines.

**Table 7:** Effect of crop forcing and water status on the anthocyanin profile of Tempranillo wines.

Compound	Year	Treatment										
		C-NF	C-F	RI-NF	RI-F	F*I	NF	F	*F*	C	RI	*I*
**MvG**	**2017**	88.3	115.9	129.9	124.4	n.s.	109.1	120.2	n.s.	102.1	122.9	*
	**2018**	141.0	202.5	144.1	178.8	n.s.	142.6	190.7	**	171.7	173.3	n.s.
	**2019**	88.9 b	150.9 a	118.0 ab	137.8 a	*	103.5	144.3	***	119.9	134.4	n.s.
**PtG**	**2017**	13.8	24.3	21.1	27.8	n.s.	17.5	26.1	**	19.0	22.7	n.s.
	**2018**	32.9	61.9	24.0	53.4	n.s.	28.5	57.6	***	47.4	42.9	n.s.
	**2019**	16.0 b	40.6 a	23.2 b	37.3 a	*	19.6	39.0	***	28.3	31.9	n.s.
**DpG**	**2017**	9.1	21.7	14.0	24.9	n.s.	11.5	23.3	**	15.4	17.8	n.s.
	**2018**	31.6	77.2	17.0	65.9	n.s.	24.3	71.6	***	54.4	47.1	n.s.
	**2019**	18.0	52.7	18.7	46.7	n.s.	18.4	49.7	***	35.4	35.7	n.s.
**PnG**	**2017**	3.4	8.0	3.4	6.9	n.s.	3.4	7.4	***	5.7	5.7	n.s.
	**2018**	23.1	36.6	8.4	22.0	n.s.	15.7	29.3	***	29.8	22.5	***
	**2019**	4.2	13.1	3.4	10.8	n.s.	3.8	12.0	***	8.7	8.3	n.s.
**CyG**	**2017**	0.6 c	1.8 a	0.8 c	1.5 b	**	0.7	1.7	**	1.2	1.3	n.s.
	**2018**	5.1	11.5	1.5	5.7	n.s.	3.3	8.6	***	8.3	6.5	**
	**2019**	2.3	6.5	2.2	5.5	n.s.	2.2	6.0	***	4.4	4.4	n.s.
**∑G**	**2017**	115.2	171.7	169.2	185.6	n.s.	142.2	178.7	*	143.5	170.5	*
	**2018**	233.7	389.6	195	325.8	n.s.	214.3	357.7	***	311.7	292.3	n.s.
	**2019**	129.4 b	263.8 a	165.5 b	238.0 a	*	147.4	250.9	***	196.6	214.7	n.s.
**MvA**	**2017**	2.9	2.9	5.8	4.1	n.s.	4.3	3.5	n.s.	2.9	4.3	**
	**2018**	3.4	5.5	3.0	4.8	n.s.	3.2	5.1	**	4.4	4.2	n.s.
	**2019**	4.8	6.3	5.9	4.6	n.s.	5.3	5.5	n.s.	5.6	6.1	n.s.
**PtA**	**2017**	0.3 c	1.3 b	2.2 a	1.6 b	***	1.3	1.5	n.s.	0.8	1.8	***
	**2018**	2.4	3.2	2.3	3.0	n.s.	2.4	3.1	**	2.8	2.8	n.s.
	**2019**	2.4	3.8	3.4	3.3	n.s.	2.9	3.5	n.s.	3.1	3.6	n.s.
**DpA**	**2017**	1.2	1.3	2.0	1.7	n.s.	1.6	1.5	n.s.	1.3	1.6	**
	**2018**	1.9	3.2	1.5	3.2	n.s.	1.7	3.2	***	2.5	2.3	n.s.
	**2019**	2.3	4.8	3.3	5.1	n.s.	2.8	4.9	*	3.5	4.0	n.s.
**PnA**	**2017**	0.4 b	0.5 b	0.7 a	0.6 ab	**	0.5	0.5	n.s.	0.4	0.6	**
	**2018**	1.4	1.5	1.4	1.1	n.s.	1.4	1.3	n.s.	1.4	1.4	n.s.
	**2019**	2.3	1.6	2.8	1.6	n.s.	2.5	1.6	n.s.	1.9	2.2	n.s.
**CyA**	**2017**	0.8	0.8	0.9	0.8	n.s.	0.8	0.8	n.s.	0.8	0.9	n.s.
	**2018**	0.3	0.6	0.3	0.6	n.s.	0.3	0.6	*	0.5	0.5	n.s.
	**2019**	1.3	2.9	1.2	3.3	n.s.	1.2	3.1	***	2.1	2.0	n.s.
**∑A**	**2017**	5.5 b	6.8 b	11.7 a	8.8 ab	*	8.6	7.8	n.s.	6.1	9.2	**
	**2018**	9.5	14.0	8.5	12.8	n.s.	9.0	13.4	**	11.7	11.2	n.s.
	**2019**	13.0	19.4	16.6	17.9	n.s.	14.8	18.6	n.s.	16.2	18	n.s.

Treatments: C-NF (no forcing and full irrigation); C-F (forcing and full irrigation); RI-NF (no forcing and deficit irrigation) and RI-F (forcing and deficit irrigation). Each value represents the mean of 8 samples (4 blocks, 2 replicates)

Parameters: Malvidin-3-glucoside (MvG); Petunidin-3-glucoside (PtG); Delphidin-3-glucoside (DpG); Peonidin-3-glucoside (PnG); Cyanidin-3-glucoside (CyG); Total monoglucoside forms (∑G); Malvidin-3-glucoside acetate (MvA); Petunidin-3-glucoside acetate (PtA); Delphidin-3-glucoside acetate (DpA); Peonidin-3-glucoside acetate (PnA); Cyanidin-3-glucoside acetate (CyA); Total acetyl-glucoside forms (∑A); Malvidin-3-glucoside coumarate (MvC); Petunidin-3-glucoside coumarate (PtC); Peonidin-3-glucoside coumarate (PnC); Cyanidin-3-glucoside coumarate (CyC); Total coumaroy-glucoside forms (∑C); Total malvidin derivates (∑Mv); Total petunidin derivates (∑Pt); Total delphinidin derivates (∑Dp); Total peonidin derivates (∑Pn); Total cyanindin derivates (∑Cy).

Statistical analysis: Different letters indicate the existence of statistically significant differences between treatments; n.s. indicates not significant; (*) significant at 5% level; (**) significant at 1% level and (***) significant at 0.1% level.

These changes in the values of phenolic compounds of the wines had an impact on their chromatic characteristics. According to the results shown in **Table 6**, F wines had higher C-Ant values (%) than NF wines in the 3 years of the study, and this trend was observed when RI were compared with C wines. Moreover, the CI values registered in the F and RI wines were higher than in the NF and C wines. It should be noticed that only F had a significant effect. Therefore, the C-Ant (%) and CI values followed the order RI-F>C-F>RI-NF>C-NF. No significant changes were observed in color composition (calculated as % 420, % 520, and % 620). Differences were found in the values of these percentages between C-NF and RI-NF wines in the 2019 vintage only. Finally, the Hue values of the RI wines tended to be lower than those found in the C wines, with significantly different values in the 2017 and 2019 vintage.

#### 3.3.3 Anthocyanin profile of Tempranillo wines

The amounts of the different anthocyanin substances (mg/L) found in the wines from the different treatments in 2017–2019 years are indicated in **Table 7**. As previously described, the anthocyanins present in wines were identified as the mono-glucoside forms (G) and acetyl-glucoside forms (A) of delphinidin (Dp), cyanidin (Cy), petunidin (Pt), peonidin (Pn), and malvidin (Mv) and the p-coumaroyl-glucoside forms (C) of Mv, Pt, Pn, and. Cy. Regardless of year, mono-glucosides were always higher than acetyl-glycosides and acetyl-glycosides higher than acetates, with Mv derivates the most abundant in all samples. The major compound was MvG in all samples analyzed, with amounts (mg/L) ranging from 88.3, (C-NF, 2017), to 202.5 (C-F, 2018), while the lowest values corresponded to PtA (0.3 in C-NF, 2017), and CyA (0.3 in C-NF, 2018 and 1.3 in RI-NF, 2019). With respect to anthocyanidin derivates, each year in the NF wines and in 2017 in the F wines the sequence was ∑Mv>∑Pt>∑Dp>∑Pn>∑Cy, and in the F wines in 2018 and 2019 it was ∑Mv>∑Dp>∑Pt>∑Pn>∑Cy. Since, no significant interactions were found when the effects of crop forcing and irrigation systems were investigated on individual anthocyanin compounds, the effects of crop forcing and irrigation were also analyzed separately (**Table 7**). The significance of the effect of F on individual anthocyanin compounds varied depending on the year, with statistical significance observed seven times in 2017, 11 in 2018, and 10 in 2019. Also, each year significantly higher values of the mono-glucoside forms were found in the F compared to the NF wines, with the exception of MvG in 2017, and therefore higher values of ∑G were found in F compared to NF wines in all study years. Although, in general, the values of acetyl-glucoside forms were higher in F than in NF, ∑A was only significant in 2018. Finally, an opposite trend was observed in the coumaroyl-glucoside forms, with significant decreases in ∑C found when F wines were compared with NF wines. Additionally, the values of ∑Mv, ∑Pt, ∑Dp, ∑Pn, and ∑Cy were higher in F than in NF wines each year, with the exception of ∑Mv and ∑Pt in 2017. It should also be noted that the magnitude of the increase (in %) in ∑Dp and ∑Pt compounds was higher than in the other compounds. Finally, it is also of interest to note that, in general, although the interaction had no statistical significance, the increases were higher in C than in RI wines in 2017 and 2019, while in 2018 the opposite trend was registered. Thus, in 2017 and 2019 the increases found in Dp were 123% and 153% in CF wines compared to C-NF, and 67.3% and 109.1% in RI-F compared to RI-NF wines. However, in 2018 Dp increases were 112.8% and 237.8% in RI-F vs. RI-NF and C-F vs. C-NF. The effect of irrigation strategy depended on the year, the compound, and the crop forcing treatment, with a significant effect in seven individual anthocyanin compounds in 2017, three in 2018, and none in 2019. In 2017, higher values were found in RI than C wines, with higher increases (in %) in NF than F wines being the general trend. Significant increases were found in every anthocyanin form and in every derivate. The highest increases were found in the values of PtA (633.3%), MvA (100%), PtC (91.9%), and PtG (52.9%) of the RI-NF wines compared to the C-NF wines. In 2018, as a general trend, lower values were found in RI compared to C, with decreases (in %) slightly higher in NF wines, although the decreases were only significant in CyG, PnG, and PnC. Finally, in 2019, the irrigation treatment did not cause changes in the values of any anthocyanin substance present in either NF or F wines. The effect of irrigation depended on the crop forcing treatment, with a trend to increased values of ∑Mv, ∑Pt, and ∑Dp observed in RI-NF compared to C-NF. However, the opposite trend was detected when C-F and RI-F were compared. In consequence, in 2017, the highest values of ∑Mv were found in RI-NF (170.7), of ∑Pt (33.5) and Dp (26.6) in RI-F, and of Pn and Cy in C-F. The following year, 2018, the maximum values of all anthocyanidin groups were registered in C-F. Finally, in 2019, the maximum values of Mv, Pt, Dp, Pn, and Cy corresponded to the C-F treatment, with very close values registered in RI-F. The minimum values of all the anthocyanidin derivates were registered in NF treatments; more specifically, in C-NF in 2017, Mv in C-NF, and the rest in RI-NF in 2018, and Mv, Pt, and Dp in C-NF and Pn and Cy in RI-NF in 2019.

### 3.4 Classification of wines; classification parameters

Multiple factor analysis (MFA) is a useful statistical method to analyze the similarities and discrepancies between a set of observations explained by data tables of different parameter sets and can also be used to explore the correlation between these parameter sets (Escofier et al., 1994). In this study, MFA was performed on the data matrices of the yield (Yield and BW), acid (TAR, MAL, TA and pH), phenolic (TPP, Ant, Cat and Tan), and chromatic (%C-Ant, CI, and Hue) group parameters. In addition, meteorological parameters (maximum temperature and SWP) were used as supplementary (non-active) variables to further explore the influence of both temperature and SWP on the composition of grapes and wines. The MFA allowed, on the one hand, exploring the differences and similarities between the different wines due to the treatment applied to the vines and, on the other hand, to know which set of variables can be used as markers of the wines from the different treatments.

**Figure 2:**
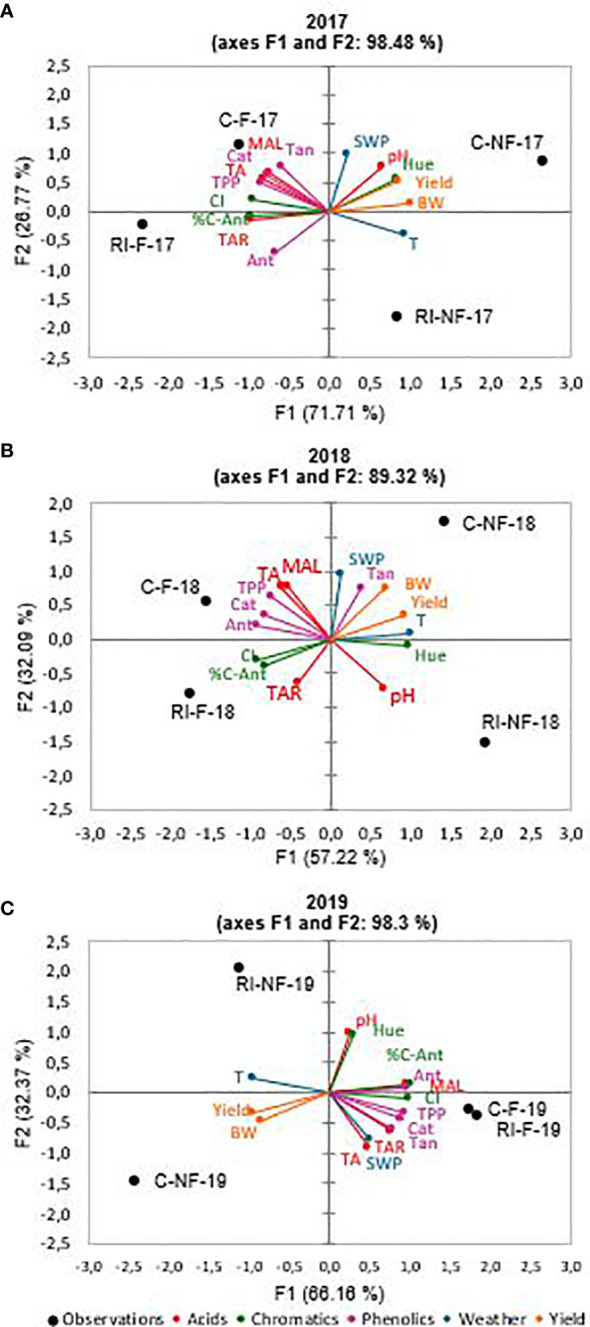
Multiple factorial analysis (MFA). **(A)** 2017; **(B)** 2018; **(C)** 2019. Acids parameters: total acidity (TA), malic acid (MAL), tartaric acid (TAR) and pH. Phenolics parameters: total polyphenolic content (TPP), anthocyanins content (Ant), tannins content (Tan) and catechins contents (Cat). Chromatics parameters: color intensity (CI), Hue and color-anthocyanins (C-Ant). Weather parameters: temperature (T) and stem water potential (SWP). Yield parameters: yield and berry weight (BW).

**Figures 2A–C** show the results of the MFA carried out on the results of the wines from the 2017, 2018, and 2019 vintages, respectively. In all years, a good variance explanation was achieved (98.5%, 89.3%, and 98.5% in 2017, 2018, and 2019, respectively). In all years, the wines were distributed along the horizontal axis F1 (which explained 71.7%, 57.2%, and 66.2% of the total variance) and were grouped according to wines from forced (F) or non-forced (NF) vines. On the other hand, the vertical axis F2, which explained 26.8%, 32.1%, and 32.4% of the total variance, separated the samples according to irrigation treatment. In 2017, NF and F samples were located on the positive and negative side of F1, respectively. According to variable contributions (%) showed in **Table 8**, this axis was defined by the yield (25.0%), acid (27.1%), phenolic (20.8%), and chromatic (27.0%) group parameters. Thus, all groups of variables contributed to a similar extent to differentiate the F from the NF samples. Almost all of the individual variables were related to higher values in F wines, with the exception of Hue, pH, yield, and BW, compared to NF wines. However, F2 was mainly associated with phenolic (43.5%) and acid (35.6%) groups of variables. Thus, the F2 axis allows distinguishing the C samples from the RI samples. The latter, located on the negative side of F2, were defined mainly by higher Ant values. In 2018, the distribution of samples on the plane defined by F1 and F2 was similar to that of the previous year. F1 grouped the samples by crop forcing and F2 by irrigation strategy. As **Table 8** reflects, in 2018, the yield (24.7%), phenolic (27.8%), and chromatic (31.6%) parameters group contributed with similar values to F1, but the acid parameters group with 16.0% only. Thus, F samples were located on the negative side of F1 and NF on the positive side (**Figure 2B**). The latter were associated to higher BW, Tan, yield, Hue, and pH. As in 2017, C and RI samples were located on the positive and negative side, respectively, of the F2 axis. RI samples were mainly defined by higher TAR and pH values. Finally, as **Table 8** shows, in 2019, all groups of variables contributed to the same extent to F1. This axis grouped NF (C and RI) wines on the negative side. These samples were defined by higher yield and BW. It should be noted that F2 differentiated RI-NF and C-NF by pH and Hue values (higher in RI-NF), and, also in 2019, RI-F and C-F were closely located on the F2 axis. Therefore, in all years of the trial, F (C and RI) wines, elaborated from vines with lower yield and smaller berries than NF, were distinguished by higher amounts of MAL, TAR, TA, TPP, Ant, Cat, CI, and % C-Ant than NF wines. In addition, RI-F wines were characterized by the maximum values of TAR and TA and C-F wines by the maximum value of polyphenolic substances. On the other hand, in 2017 and 2019, RI (F and NF) wines, from RI with lower production and smaller berries than C vines, and higher SWP were characterized by lower pH and Hue values than C wines.

**Table 8:** Variable contributions (%) to multiple factorial analysis (MFA).

Parameter	Year					
	2017		2018		2019	
	F1 axe	F2 axe	F1 axe	F2 axe	F1 axe	F2 axe
**TAR**	9.9	0.6	2.1	8.8	6.9	9.7
**MAL**	5.9	11.4	3.6	13.4	10.8	0.4
**TA**	6.9	8.3	4.9	13.1	2.5	18.7
**pH**	4.4	15.3	5.4	10.8	0.7	22.4
**Acids parameters**	**27.1**	**35.6**	**16.0**	**46.2**	**20.9**	**51.1**
**TPP**	7.1	6.1	6.9	8.6	7.2	1.8
**Ant**	4.2	12.4	10.8	0.9	7.6	0.1
**Cat**	6.0	9.5	8.3	3.0	6.5	3.3
**Tan**	3.5	15.4	1.7	13.1	5.0	6.3
**Phenolics parameters**	**20.8**	**43.5**	**27.8**	**25.6**	**26.2**	**11.4**
**%C-Ant**	10.0	0.2	8.8	3.5	13.7	0.5
**CI**	9.9	1.1	10.7	2.2	13.5	0.3
**Hue**	7.1	8.8	12.1	0.2	1.2	26.2
**Chromatics parameters**	**27.0**	**10.1**	**31.6**	**5.9**	**28.4**	**27.0**
**Yield**	10.5	10.3	16.1	4.2	13.3	3.5
**BW**	14.5	0.6	8.6	18.1	11.2	6.9
**Yield parameters**	**25.0**	**10.8**	**24.7**	**22.4**	**24.5**	**10.5**

Acids parameters: Total Acidity (TA), Malic acid (MAL), Tartaric acid (TAR) and pH. Phenolics parameters: Total polyphenolic content (TPP), Anthocyanin content (Ant), Tannin content (Tan) and Catechin contents (Cat). Chromatics parameters: Color intensity (CI), Hue and Color-Anthocyanins (C-Ant). Weather parameters: Temperature (T) and Stem water potential (SWP). Yield parameters: Yield and Berry weight (BW).

**Figure 3:**
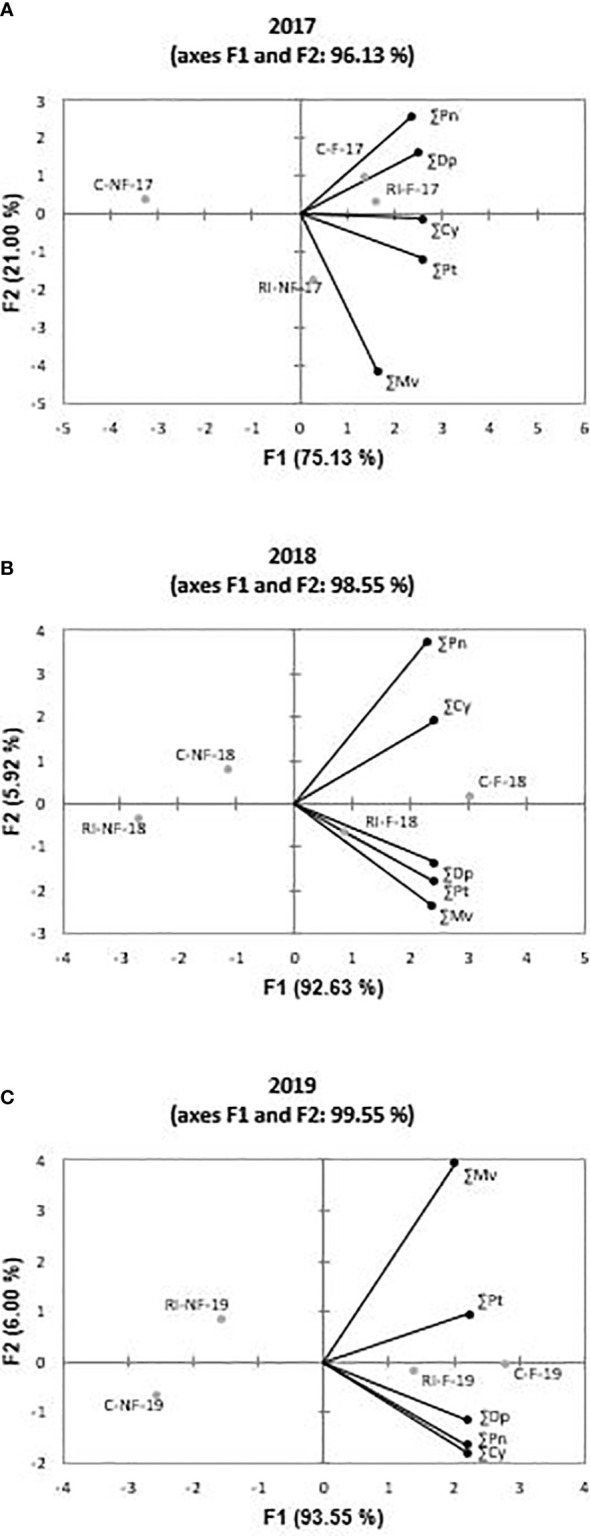
Principal components analysis on anthocyanin profile of Tempranillo wines: **(A)** 2017; **(B)** 2018;**(C)** 2019. Malvidin (∑Mv), petunidin (∑Pt), delphinidin (∑Dp), peonidin (∑Pn) and cyanidin (∑Cy). Treatments: C-NF (no forcing and full irrigation); C-F (forcing and full irrigation); RI-NF (no forcing and deficit irrigation) and RI-F (forcing and deficit irrigation).

PCA was performed with the values of ∑Mv, ∑Dp, ∑Pt, ∑Pn, and ∑Cy of each year to determine general trends in the different wines. **Figures 3A–C** shows the distribution of samples in 2017, 2018, and 2019, respectively. PC-a, PC-b and PC-c accounted for more than 95% of the total variance. F1 axe covered 75% in 2017 and about 90% in the following years. From these figures, it is clear that, with the exception of RI-NF in 2017, the wines can be grouped in NF and F. These last samples were related with higher values of all derivate compounds with the exception of ∑Mv in 2017.

## 4 Discussion

The dates of the most relevant phenological stages were modified in the forced treatments compared to the non-forced vines (**Table 2**). This implied that temperatures were also different during the ripening period (veraison to harvest), with cooler maximum and minimum temperatures in the forced treatments (**Figure 1**). This lower evapotranspirative demand during berry ripening in forced vines was also reflected in the vine water status for which, regardless of the irrigation strategy applied, water stress during ripening (from veraison to harvest) was milder than in the non-forced treatments, with SWP values ranging between −0.58 MPa and −0.62 MPa and between −0.67 MPa and −0.77 MPa in C-F and RI-F, respectively. In this trial, coinciding with that reported by Gu et al. (2012) in cv. Cabernet Sauvignon and by Martinez De Toda et al. (2019) in cv. Tempranillo, the application of forcing, regardless of the irrigation strategy, caused a delay in the harvest date and decreases in BW and yield. The factors responsible for the production penalty are the number and weight of berries in the forced treatments. The displacement of the phenological cycle due to crop forcing places the new flowering and berry growth between July and August, coinciding with a period of higher temperatures (**Figure 1**). According to Kliewer and Antcliff (1970), both the lower fruit set rate and the decreases in berry weight could be attributable to increases in temperature during flowering and fruit set and in the ambient light regime in the microclimate around the clusters. This could influence the berry sink capacity (i.e. the ability to attract photoassimilates) in F berries compared to NF berries. The yield decrease in RI treatments compared to C treatments may have been due to the worse water status of the RI vines, both in the pre-veraison period with a mild level of stress and in the post-veraison period with a more severe level as shown by the SWP values recorded during the 2017-2019 seasons (**Table 3**) (Grimes and Williams, 1990; Intrigliolo and Castel, 2010; Santesteban et al., 2011; Intrigliolo et al., 2012; Pérez-Álvarez et al., 2021). The intensity of this effect may vary with variety (Mirás-Avalos and Intrigliolo, 2017), but in cv. Tempranillo, previous work carried out in this same vineyard reported a significant decrease in final berry size and yield at harvest when pre-veraison water stress was applied (Valdés et al., 2009; Uriarte et al., 2015; Mancha et al., 2021). In this regard, it should be considered that the degree of pre-veraison water stress that can be naturally induced depends on the soil water available at flowering, which in turn depends on previous rainfall levels and the water used by the vine during the spring (Lopes et al., 2011). Again, the change in phenology due to forcing shifted the pre-veraison period later in the season, when soil water content had possibly already decreased significantly. This allowed lower SWP to be reached in the forcing vines during pre-veraison throughout the three study seasons (**Table 3**) and, consequently, contributed to the yield penalty and lower berry weight observed in the forcing treatments (C-F and RI-F).

Acidity is not only important for flavor balance and the organoleptic properties of wine, but also contribute to wine stability (Van Leeuwen and Darriet, 2016). Malic and tartaric acids are the most common organic acids in grapevine fruit and they are the determinants of the TA of berries and wines. Normally, both acids reach their highest concentrations near veraison. In the second phase of growth, termed ripening, metabolite concentrations increase or decrease depending on net biosynthesis or metabolization and growth dilution, both mechanisms being genotype-dependent (Dai et al., 2011; Keller et al., 2016). It is believed that, once synthesized, tartaric acid remains stable and that the content does not vary in terms of quantity per fruit during ripening (Terrier et al., 2001; Rösti et al., 2018). Malic acid, however, is metabolized and used as an energy source during the process (Rienth et al., 2016). Therefore, tartaric acid concentration decreased in the samples of must and wines from the more irrigated treatments which resulted in the highest berry weight (C-NF). It is known this acid is less affected than malic acid by environmental conditions (Ruffner, 1982), and thus its concentration was probably more determined by the dilution effect in must as well as by increased precipitation of bitartrate potassium salts (Iland and Coombe, 1988). In our case, the lower temperatures during the ripening period of the F berries impeded the combustion of this acid in NF samples. Torres et al. (2017) reported that in cv. Tempranillo the extent of alteration in primary metabolism due to temperature was higher than in secondary metabolism, which was mainly affected by deficit irrigation. The effect of water stress during the herbaceous period of berry development on acidity has been reported for cv. Tempranillo and other varieties (Salón et al., 2005; Girona et al., 2009; Intrigliolo and Castel, 2010). Must and wines from F were more acid mainly because of the much larger concentration of malic acid than in the NF samples, especially in RI treatments. This organic acid was the main contributor to changes of acidity (García Romero et al., 1993). In this work, in general, the highest TA values were found in in C-F samples (must and corresponding wines). These results are a consequence of one hand, of the increase in the synthesis of malic and tartaric acid due to higher assimilation rates (Esteban et al., 1999; De Souza et al., 2005; Salón et al., 2005). On the other hand, the lower malic acid respiration rate decreased by the lower temperatures reached by the clusters less exposed to sunlight as a result of the increase in leaf area in C irrigation practices due to higher vegetative growth (Spayd et al., 2002; De Souza et al., 2005). Our findings are in agreement with those of previous works that independently analyzed the effect of forcing (Martinez De Toda et al., 2019) and reduced deficit irrigation (Uriarte et al., 2016) on cv. Tempranillo. In this regard, it should be noted that the present study is the first to report that the better climatic conditions and temperatures during the ripening of RI-F samples mitigated the decrease caused by the RI strategy in NF wines, with TA values of RI-F wines always close to those found in C-F wines. Since malic is a weaker acid than tartaric (i.e. malic acid has higher pKa and dissociates incompletely), unfortunately, the effect of crop forcing on wine pH was only slight. These results reduce the success of the crop forcing technique because one of the main problems, particularly pronounced in Tempranillo wines, of current oenology is the high pH of wines.

Temperature, water status drought, and light intensity are factors of influence in the synthesis, accumulation, and concentration at harvest of phenolic substances of grapes and, in consequence, of the wines that are elaborated (Arrizabalaga et al., 2018; Del-Castillo-Alonso et al., 2021; Pérez-Álvarez et al., 2021; Valdés et al., 2022). Since, in this work, crop forcing and irrigation modified the temperature (**Figure 1**) and water status during the vegetative period (**Table 3**) and the yield and berry weight to different extents according to year and treatment (**Table 4**), the initial C-F, RI-F, and RI-NF mashes displayed different phenolic content than C-NF and, in consequence, the content of polyphenolic families and the chromatic characteristics of the wines were also modified. The increase in anthocyanin content in F samples (initial mashes and in the respective wines) could be associated with the lower temperatures registered during the ripening cycle of these treatments than in the NF samples (**Figure 1**). It is known that as summer temperature rises to atypical values, the anthocyanin biosynthetic genes are downregulated, reducing berry skin anthocyanin biosynthesis (Conde et al., 2016). Arrizabalaga et al. (2018) showed in different Tempranillo clones that elevated temperature reduced anthocyanin concentration. They reported that, with the same °Brix, the anthocyanin concentration was lower at 28°C/18°C than 24°C/14°C, indicating a decoupling effect of elevated temperature during berry ripening explained by changes in the relative rate of response of anthocyanin and sugar build up, rather than delayed onset of anthocyanin accumulation. These authors also referenced the inhibition of mRNA transcription of the anthocyanin biosynthetic genes, as well as chemical and/or enzymatic degradation of the anthocyanins by the high temperatures reported in previous works (Mori et al., 2007). Furthermore, temperature may also reduce the anthocyanin content, affecting its subcellular transport through the down-regulation of several transmembrane transporter-encoding genes involved in the import of anthocyanins in the vacuole (Carbonell Bejerano and Martinez Zapater, 2013). With respect to NF and C, in both F and I samples the increase in anthocyanins is also associated with a berry-size concentrating effect associated with a decrease in BW and lower vine vigor (Cortell et al., 2007; Pérez-Álvarez et al., 2021; Aris et al., 2022; Valdés et al., 2022). Additionally, in the RI effect, the up-regulation of the biosynthetic pathway caused by water stress should be considered (Castellarin et al., 2007). It is known that the release of anthocyanin and tannin compounds from berry skins into the wine is affected by several viticulture and oenological practices (Guidoni & Hunter, 2012). In this work, since in terms of Ant, the wines reflect the trend observed in the initial mash, our results suggest that the extractability of anthocyanin from the grapes was scarcely modified by the treatments. However, in the case of tannins, in 2017 while no significant differences were found in the tannin content of the initial mash due to forcing and irrigation, they were found in the wines. In 2018, lower values (*p* <0.05) were found in the RI must but not in RI wines compared to their respective C samples. These results need to be confirmed in future research.

Since acylated and coumarate derivates are considered to be among the most stable compounds (Ortega-Regules et al., 2006), and Cy, Dp, and Pt derivates are more sensitive to enzymatic oxidation (except for laccase) and non-enzymatic oxidation (catalyzed by copper or iron ions) to produce o-diquinones, or even o-diphenol dimers than Mv and Pn (Jackson, 2008), the anthocyanin profile determines the color and stability of wines. It is therefore essential to examine the effect of the techniques applied on the anthocyanin profile of the wines produced. While the extent of the change depended on the derivate considered, crop forcing modified the anthocyanin profile of the wines elaborated. Regardless of irrigation strategy, the highest increases were registered in Dp anthocyanidin. In this regard, Tarara et al. (2008) reported that lower temperatures were associated with increases in Dp, Cy, Pt, and Pn derivates, but found no influence on concentrations of Mv derivates. This behavior was only found in our study in 2017 because ∑Mv increased in 2018 and 2019. Otherwise, Mv and Pn compounds are more resistant to oxidation, than Cy, Dp, and Pt. When the HS/LS (high sensitivity/low sensitivity) ratio was calculated as ∑(Cy + Dp + Pt)/∑(Mv + Pn), the mean values reached 0.68 in C-NF, 0.85 in C-F, 0.55 in RI-NF, and 0.83 in RI-F. The order RI-NF<C-NF< RI-F<C-F was observed in all years. Thus, F wines were more sensitive to oxidation than NF wines and, for a given crop forcing treatment, RI wines more than their respective C wines. The different wine profiles are a consequence of the changes found in the anthocyanin profiles of the grapes (results not shown) and the different rates and amounts of individual anthocyanin extraction from skins into wine and their transformation during fermentation (Guidoni and Hunter, 2012).

Along with anthocyanins, catechins and tannins can strongly impact the quality of red wines *via* their contributions to wine bitterness and astringency (Cheynier et al., 1997; Vidal et al., 2003; Kennedy et al., 2006). Our results are in agreement with those of other studies that showed a reduced response of tannins to irrigation treatments (Ollé et al., 2011). The increase of these compounds in F wines (C and RI) may be attributable to high temperatures impairing tannin synthesis of the berries and also the degree of galloylation at the transcriptomic levels, as described by Rienth et al. (2016). According to Bonada et al. (2015), temperature could affect tannin extractability from seed or skin indirectly by uncoupling berry seed and skin development and modifying the number of seeds or skin mass per berry. In this last regard, F grapes had a higher percentage of seeds in the fresh berry weight than NF grapes (4.6 and 4.0, respectively, as global interannual mean).

With respect to C-NF, these changes may modify the phenolic profile evolution of the wines elaborated and their color stability and their consequent chromatic characteristics. Since F and RI treatments caused a loss of yield it would be interesting to elaborated blend wines.

Many studies have used statistical techniques to find correlations between phenolic compounds and color parameters during the maturation and ageing processes of red wine (Gamero et al., 2018). In one interesting work Monagas et al. (2006) showed that chromatic attributes of red wines could be predicted by their phenolic profile using polynomial regression techniques. The substances which provided the best fitting model in that study were the anthocyanin compounds. In addition, when Gamero et al. (2014) investigated the correlations between the phenolic composition and the chromatic characteristics of Tempranillo wines, they found that CI was high and positively correlated with the presence of G, C, Dp, Mv, and Pt. In consequence CI was higher in F than in NF wines. Crop forcing modified the maximum temperature during the vegetative period. This parameter, considered as supplementary variable in MFA, was significant and strongly correlated to F1 axe. The irrigation strategies modified the SWP parameter correlated with F2 axe. Since F1 axe explained 71.7%, 57.2%, and 66.2% of the variation, and the location of samples was similar all years, it implies that the temperature during the vegetative period had a strong effect and a consistent response. Our results are in agreement with previous research in that Torres et al. (2017) reported a good separation of grape samples grown at different temperatures mainly based on differences in TAR. When Arrizabalaga et al. (2018) employed a PCA on plants grown at 24°C/14°C and 28°C/18°C, the first two principal components explained about 75% of the total variability and clearly separated samples according to the temperature regime. F2 axe explained a lower percentage of the variance of F1. Therefore, the impact of irrigation strategy was lower and varied with the year considered. It is noticed that while the contribution of the each parameter group to F1axe was similar in the 3 years under study the contributions to F2 axe depended on the year considered, with NF samples more affected by the irrigation strategy than F samples. When Bonada et al. (2015) subjected to PCA the chemical and sensory profiles of Shiraz grape wines produced from vines exposed and not exposed to hydric and thermal stress, they found that F1 axe explained ∼53% of the variation and was a function of the temperature treatment, with the remaining 37% explained by F2 and F3 axes, which were related to the water treatment. According to these authors, those differences suggest a comparatively higher impact of temperature than water on grape and wine composition. Thus, it can be concluded that, in line with Arrizabalaga et al. (2018), the extent of alteration in primary metabolism due to temperature was higher than in secondary metabolism, which was mainly affected by deficit irrigation. Finally, it has been noted that berry weight did not constitute a determining factor in wine composition. In this sense, these results support the findings of Walker et al. (2005) and Matthews & Nuzzo (2007) who argued that the viticultural practices used to control yield in a vineyard are more important than the yield values per se in determining the quality of the resulting grapes and wines, and that the environmental conditions determining berry size are more important than the size per se in determining the quality of the grapes and resulting wines.

## 5 Conclusions

The impact of climate change factors requires the use of direct short-term methods that involve changing environmental factors. This research provides evidence of changes in the composition of Tempranillo cv. wines in response to temperature and water status of vines during the vegetative period. The significance and extent of the temperature impact was higher and more consistent than that of water status. Wines from forced vines (elaborated with berries grown at lower temperature) had, in general, higher values of total acidity, malic acid, anthocyanins, catechins, and total polyphenols. In addition, color intensity and co-pigmented anthocyanin contents were higher in these wines. However, wines from forced vines were more sensitive to oxidation than wines from non-forced vines. Moreover, the significance and extent of the response to crop forcing was more consistent than irrigation across the different vintages. Thus, these results indicate that adaptation to climate change in south Mediterranean Europe might be plausible with the application of crop forcing. However, due to the decrease in yield, wines from crop forcing could be used as “good modifiers” of wines from vines grown with conventional techniques to improve their acid and phenolic composition as well their chromatic characteristics.

## Data availability statement

The raw data supporting the conclusions of this article will be made available by the authors, without undue reservation.

## Author contributions

Conceptualization, NL, DM, and MEV; Data curation, NL, MP, DM, and MEV; Formal analysis, NL, MP, DM, LM, DU, and MEV; Funding acquisition, MP; Investigation, NL, MP, DM, LM, DU, and MEV; Methodology, NL, MP, DM, LM, DU, and MEV; Project administration, MP; Resources, MP, DU, and MV; Software, NL and MEV; Supervision, NL and MEV; Validation, NL and MEV; Writing, original draft, NL and MEV; Writing, review and editing, NL and MEV. All authors contributed to the article and approved the submitted version.
